# Analysis of Pressure Distribution in Transfemoral Prosthetic Socket for Prefabrication Evaluation via the Finite Element Method

**DOI:** 10.3390/bioengineering6040098

**Published:** 2019-10-24

**Authors:** Mohd Syahmi Jamaludin, Akihiko Hanafusa, Yamamoto Shinichirou, Yukio Agarie, Hiroshi Otsuka, Kengo Ohnishi

**Affiliations:** 1 Department of Bio-Science and Engineering, Shibaura Institute of Technology, Tokyo 135-8548, Japan; hanafusa@shibaura-it.ac.jp (A.H.); yamashin@shibaura-it.ac.jp (Y.S.); 2 Department of Supporting Prosthetic Orthotics, Niigata University of Health and Welfare, Niigata 950-3102, Japan; agarie@nuhw.ac.jp; 3 Department of Prosthetic and Orthotics, University of Human Arts and Science, Iwatsuki Ward 339-0077, Japan; hiroshi_otsuka@human.ac.jp; 4 Department of Science and Engineering, Tokyo Denki University, Tokyo 120-8551, Japan; ohnishi@mail.dendai.ac.jp

**Keywords:** prosthetic socket, finite element method, finite element analysis, transfemoral residuum, biomechanics, 3D model

## Abstract

In this study, we estimated and validated the pressure distribution profile between the residuum and two types of prosthetic sockets for transfemoral amputees by utilizing a finite element analysis. Correct shaping of the socket for an appropriate load distribution is a critical process in the design of lower-limb prosthesis sockets. The pressure distribution profile provides an understanding of the relationship between the socket design and the level of subject comfortability. Estimating the pressure profile is important, as it helps improve the prosthesis through an evaluation of the socket design before it undergoes the fabrication process. This study focused on utilizing a magnetic resonance imaging (MRI)-based three-dimensional (3D) model inside a predetermined finite element simulation. The simulation was predetermined by mimicking the actual socket-fitting environment. The results showed that the potential MRI-based 3D model simulation could be used as an estimation tool for a pressure distribution profile due to the high correlation coefficient value (*R*^2^ > 0.8) calculated when the pressure profiles were compared to the experiment data. The simulation also showed that the pressure distribution in the proximal area was higher (~30%) than in the distal area of the prosthetic socket for every subject. The results of this study will be of tremendous interest for fabricators through the use of a finite element model as an alternative method for the prefabrication and evaluation of prosthetic sockets. In future prosthetic socket fabrications, less intervention will be required in the development of a socket, and the participation of the subject in the socket-fitting session will not be necessary. The results suggest that this study will contribute to expanding the development of an overall prefabrication evaluation system to allow healthcare providers and engineers to simulate the fit and comfort of transfemoral prosthetics.

## 1. Introduction

Achieving a proper residuum–socket interface by ensuring an optimum distribution of the interface loads is critical to a successful prosthetic fitting and to the rehabilitation of lower-limb amputees [1]. In Japan, there has been an estimated 22.4% yearly increase in amputees over the past five years due to a high number of patients with peripheral vascular diseases [2]. The high demand for prosthetic sockets has provided an opportunity for fabricators to expand the socket manufacturing market. However, the design of a prosthetic socket is a time-consuming process, starting with measuring the subject amputee, creating a positive mold, shaping a socket, carrying out a socket-fitting session, improvising the prosthetic socket, and finalizing the socket position using a knee-joint mechanism. This process takes approximately two to three months before the socket can be used [2]. 

In conjunction with this, numerous studies have been conducted to investigate the possibility of utilizing theoretical analysis to provide an alternative evaluation for the prefabrication of a prosthetic device. For instance, investigations into the stress distribution between the residuum and prosthetic socket by utilizing a finite element analysis have shown that (although the results were promising in terms of the stability of the model geometry) the socket models were not realistically developed because the shape of the socket was similar to the residuum shape [3,4,5,6]. Thus, the geometrical changes of the residuum could not be clearly seen. In addition to focusing on the socket design and manufacturing methods, Sengeh et al. [7] investigated effects on the accuracy of the actual residuum parameter in the design of a residuum model with multiple materials. The residuum was modeled using a subject-specific magnetic resonance (MR) image to allow the model to be evaluated through a numerical approach, which inspired a modeling of the residuum with a subject-specific parameter in the present study but with a different methodology. Portnoy et al. [8] also reported that using a subject-specific real-time analysis of the internal tissue loads in the residuum is a practical tool for evaluating the internal stress inside the residuum in a clinical setting or outdoors. From another aspect, Colombo et al. [9] managed to develop a computer-aided environment, namely, a socket-modeling assistant (SMA), combining knowledge from prostheses and a 3D model simulation to create a prosthesis socket. Such research has resulted in the creation of a prosthesis socket using the additive manufacturing (AM) technique, although a compatibility analysis of the created socket has yet to be carried out.

This study focused on an investigation of the interaction pressure between a patient-specific multimaterial three-dimensional (3D) model and two types of ischial–ramal containment (IRC) sockets when they were completely donned. One of the IRC sockets used was a University of California, Los Angeles (UCLA), socket (developed at UCLA), which is a prosthetic socket that applies a contour-adducted trochanteric controlled alignment method (CAT-CAM). This socket is based on a study conducted by Sabolich and was inspired by research by Long [10]. The other IRC was a manual compression casting technique (MCCT) socket developed by Agarie. The difference between the two sockets is the following: the stability of the IRC-MCCT socket was improved through an adjustment of the anterolateral and sagittal directions of the IRC-UCLA socket [11]. In this study, an estimation of the pressure distribution during the interaction between the subject’s residuum and both sockets was investigated. The pressure distribution profile was a key factor in determining the potential deep tissue injury from a prosthetic device [12]. Many subjects experience discomfort with their sockets due to an improper fit, resulting in skin problems [13]. These issues are associated with a particular socket design. Such loading conditions, tissue stresses, and strains can be evaluated using a computational simulation [7]. Reynold et al. [14] proposed a simulation approach in modeling the contact interface between the residuum and the rectified socket. However, the geometrical changes in the residuum were ignored because the socket used in the study was rectified from the outer layer of the residuum.

Many theoretical analyses use a finite element method (FEM) as a medium in quantitatively evaluating a biomimicking model [1,5,6,7,8,11]. The FEM is a powerful tool to understand the load transfer in the interaction between human anatomy and a prosthetic device. The FEM also provides a better understanding of the effects of a socket modification on prosthetics [14,15] and offers a prediction of stress, strain, and motion at any location of a model, as well as proficient parametric studies [6]. In conjunction with this, we proposed utilizing the FEM combined with an image processing (IP) method to evaluate the estimation of the pressure distribution profile of transfemoral amputee subjects. The study was motivated by the lack of a quantitative analysis system for evaluating a prefabricated prosthetic socket. The evaluation was aimed at improving the socket design of the prosthesis according to the stress distribution profile of the individual subjects. The process can provide an opportunity for each subject to own a stress reduction prosthetic socket.

## 2. Materials and Methods

### 2.1. Residuum and Socket Model Construction

**Figure 1:**
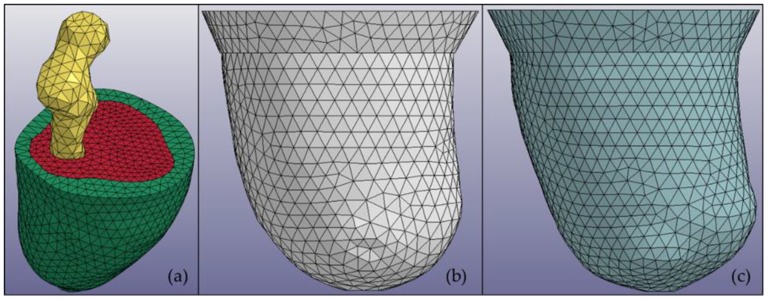
Multimaterial 3D models of (**a**) a residuum in which every part is distinguished by color: bone (yellow), muscle (red), and fat (green); (**b**) a manual compression casting technique (MCCT) socket; and (**c**) a University of California, Los Angeles (UCLA) socket.

This study focused on utilizing a subject-specific MR image to create a multimaterial residuum model. The MR images for the subjects were obtained using a Siemens Magneton Symphony Maestro class 1.5 T. The residuum model created through this study was categorized into three main parts, i.e., fat, muscle, and bone. Clouds of 30 MR images were arranged vertically layer by layer with 5-mm spacing between the images. The center point of the bone part was selected and used to create 36 trajectory lines surrounding the residuum image. The intersection points between the trajectory line and the residuum perimeter were connected by creating multiple cross-sections in a single layer. Then, every cross-section was linked to cross-sections of the other layers to construct the 3D model. The construction was made using the Swept Blend (SB) function in Creo software (PTC Ltd., Boston, MA, USA). The same procedure was applied to create socket models using different clouds of MR images. In this study, LS-DYNA software (Livermore Ltd., Livermore, CA, USA) was also used to initiate the meshing procedure for each model. Figure 1 shows an example of a subject residuum meshed model with both sockets.

The materials of the parts were modeled as isotropic with uniform elastic properties in all directions and were assumed to be homogenous with consistent material properties. Soft tissue was considered a composite material comprised of collage fibers embedded in a softer isotropic material. On the basis of previous studies [16,17,18], a viscoelastic material was chosen to represent the soft tissue material, which was formulated using a strain–energy function based on quasi-linear viscoelastic (QLV) theory. The calibration of the QLV material was conducted in a previous study and showed good agreement with the cadaver data in terms of the maximum force and displacement [18]. QLV material properties were selected because they have better biofidelity than linear elastic properties do in low-speed impact tests [18]. The soft tissue material function was formulated by Weiss [16] and is expressed through Equation (1) below:

(1) $$W=W_{1}+W_{2}+W_{3}.$$

The first term models the ground substance matrix as a Mooney–Rivlin material expressed as Equation (2):

(2) $$W_{1}=C_{1}\left(I_{1}-3\right)+C_{2}\left(I_{2}-3\right),$$

where *I*_1_ and *I*_2_ are invariants of the right deformation tensor. The second term *W*_2_ = *F*(*λ*) is defined to capture the behavior of the crimped collagen under tension, and the term is inapplicable to the materials because the direction of the fibers (muscle and fat) is not defined in the model. The role of the third term in the strain energy function is to ensure a nearly incompressible material behavior:

(3) $$W_{3}=\frac{1}{2}K{\left[\ln\left(J\right)\right]}^{2},$$

where *J* = det *F* is the third invariant of the deformation tensor, and *K* is the bulk modulus. In this study, terms 1 and 3 of the function were used to express the viscoelastic properties for the soft tissue. The reduced relaxation function for the soft tissue material *G*(*t*) is represented through a Prony series [18]:

(4) $$G\left(t\right)=\sum_{i=1}^{3}S_{i}exp\left(\frac{-t}{T_{i}}\right).$$

Two terms in the strain–energy function were used to define the reduced relaxation function of the skin, fat, and muscle due to ignorance regarding the second term because the directions of the skin, fat, and muscle model were undefined. The formulation parameters used in the simulation are listed in Table 1, where *C*, *S*, *T*, and *K* denote the hyperelastic material constants, spectral strength, characteristic time, and bulk modulus, respectively [18].

The bone and socket were categorized as a solid material. The bone was modeled using the density of the femur with a Young’s modulus (YM) of 17,700 MPa and a Poisson’s ratio (PR) of 0.3. The actual socket was designed using an acrylic plastic with a YM of 1885 MPa and a PR of 0.39.

### 2.2. Predetermined Environment for Simulation

In this study, the simulation was generated by replicating the socket fitting session. During the actual socket fitting, the subject was required to stand to accurately measure the comfort level while wearing the socket. During the simulation, 50% of the body weight of the subject was used as an indicator that the subject was standing. Because the geometrical shape of the residuum and socket were different, the distance during the donning process was taken into consideration. The ideal velocity was suggested to be 0.5 mms^−1^ and needed to be constant starting from the initial position until the residuum reached the distal end of the socket. 

The definition of contact used in the simulation was divided into two parts. The first contact between the residuum and socket was defined as surface-to-surface contact. A coefficient of friction of 0.5 was assigned as an interaction property for the contact surfaces based on that used in another study [19]. The second contact definition applied corresponded to a tied contact between the bone and muscle, which is a simple way of permanently bonding surfaces and preventing slave nodes from separating or sliding relative to the master surface. This method of contact was obtained from a previous study [20]. The definition of contact was based on the hypothesis applied to the connection between the skin and fat and the fat and muscle, in which relative motion was neglected.

**Figure 2:**
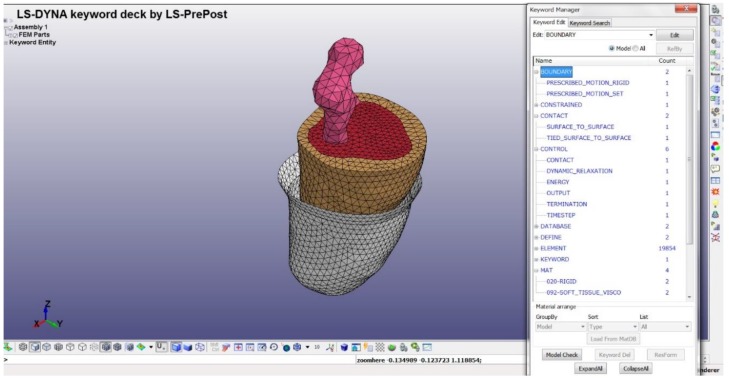
Overview of simulation environment in LS-Dyna solver. The residuum is moving vertically with 50% of the body weight located at the top of the bone. The socket is constrained in the vertical direction to allow for connectivity with the residuum when it is completely donned.

The basic principle of the simulation relied on the fact that the residuum should move toward the socket. In the residuum, 50% of the body weight was at the top to emulate a bipedal stance. Regarding the socket, the horizontal movement was constrained to realize contact between the residuum. Figure 2 shows a graphical overview of the simulation environment.

### 2.3. Experiment Analysis

**Figure 3:**
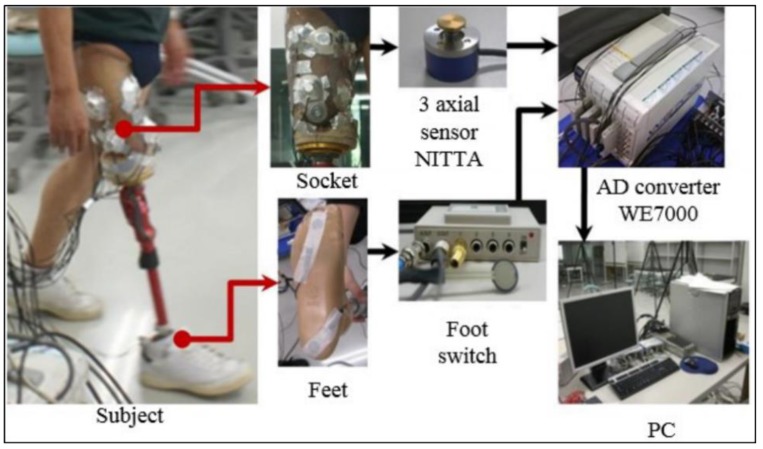
Schematic diagram of pressure analysis experiment.

The triaxial force sensors NITTA PD 3-32-05-015 [21] were used in the experiments. These force sensors can resolve the force applied to the surface in three components, two shear components in the orthogonal directions (tangential to the skin surface) and one normal stress component (normal to the skin). Eight sensors corresponding to eight areas of the socket were applied for the measurements. Four sensors were applied in four directions, i.e., the anterior, posterior, medial, and lateral directions. A schematic of the experiment is shown in Figure 3, which includes sensors on the socket of the patient, an analogue-to-digital converter, data acquisition software, and a computer. After measuring the forces, the pressure was calculated using the equation below:

(5) $$P=\frac{V}{C}\left(\frac{g}{4.5^{2}\pi 10^{-12}}\right),$$

where *g* denotes the acceleration from gravity, 4.5 denotes the radius of the sensor surface (in millimeters), *V* denotes the voltage generated, and *C* denotes the calibration coefficient.

## 3. Results

**Figure 4:**
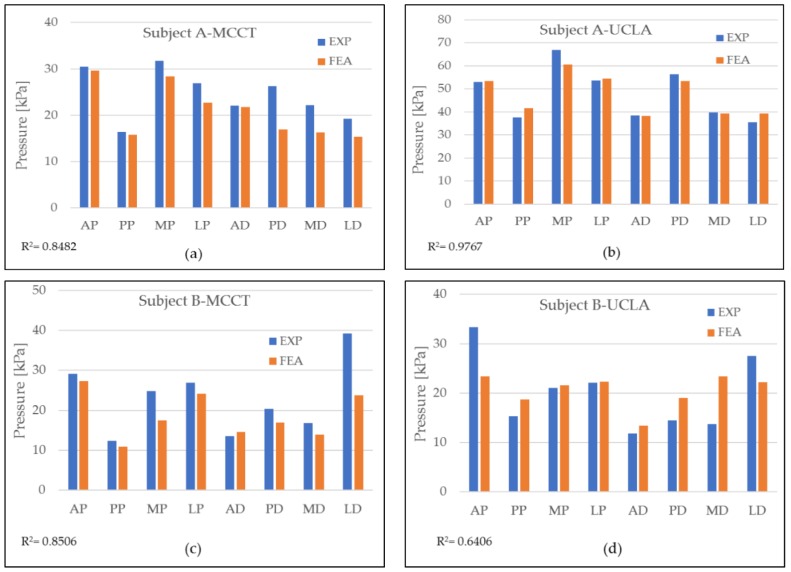
Pressure distribution during simulation (FEA) and comparison to experiment data (EXP) for each subject when wearing both sockets at eight different locations: (**a**) pressure distribution for subject A in MCCT socket; (**b**) pressure distribution of subject A in UCLA socket; (**c**) pressure distribution for subject B in MCCT socket; and (**d**) pressure distribution for subject B in UCLA socket.

In this study, a two-tetrahedral finite element model was created that corresponded to the number of subjects. Subject A was a male, 35 years of age, wearing a socket on his left leg, with seven years of history wearing a socket. Subject B, also a male, 47 years of age, was a right-leg amputee with five years of experience wearing a prosthetic socket. Both subjects were provided with two prosthetic socket models, i.e., the UCLA and MCCT sockets. For each subject, a donning simulation was applied using an LS-Dyna Solver (Livermore, Ltd., Livermore, CA, USA) as a simulator medium to observe the pressure distribution during the complete donning stage. The time of the computation varied between the subjects, but the average time was recorded as 6 h using a single Intel Xeon CPU 2.8 GHz device. During the complete donning stage, the pressure distribution was assumed to be similar to the actual pressure when wearing the socket. Figure 4 shows the results of the pressure distribution for each subject during the complete donning of the UCLA and MCCT sockets. The pressure was determined in eight locations of the residuum, i.e., the anterior proximal (AP), posterior proximal (PP), medial proximal (MP), lateral proximal (LP), anterior distal (AD), posterior distal (PD), medial distal (MD), and lateral distal (LD). A comparison to the experiment results was also made at locations similar to the sensors used in the simulation.

**Figure 5:**
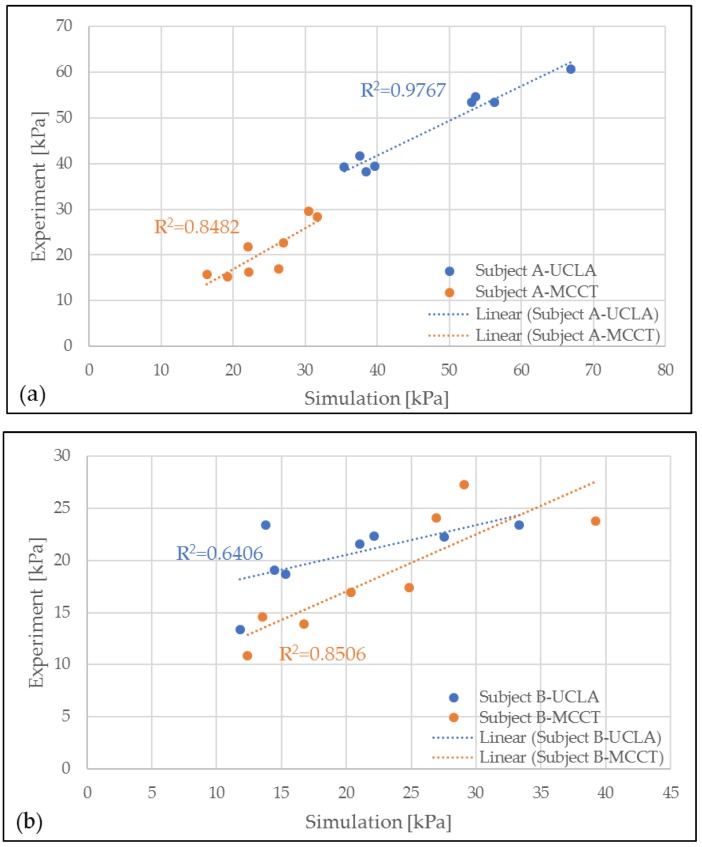
Correlation between experiment (EXP) and simulation (FEA) results for MCCT and UCLA socket for subjects (**a**) A and (**b**) B.

A validation of the simulation results was conducted by comparing the pressure in the eight locations of the residuum model to the clinical experiment data, as shown in Figure 5. A high correlation was observed throughout most of the comparison. However, in the results for subject B-UCLA, the correlation was lower than 0.8, although this was considered a positive correlation because the average error for the measurement was considered to be small (<10%).

**Figure 6:**
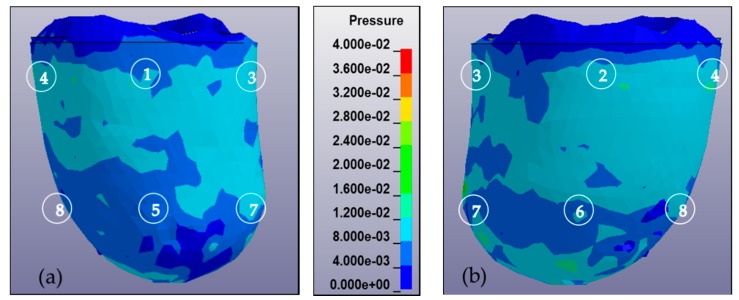

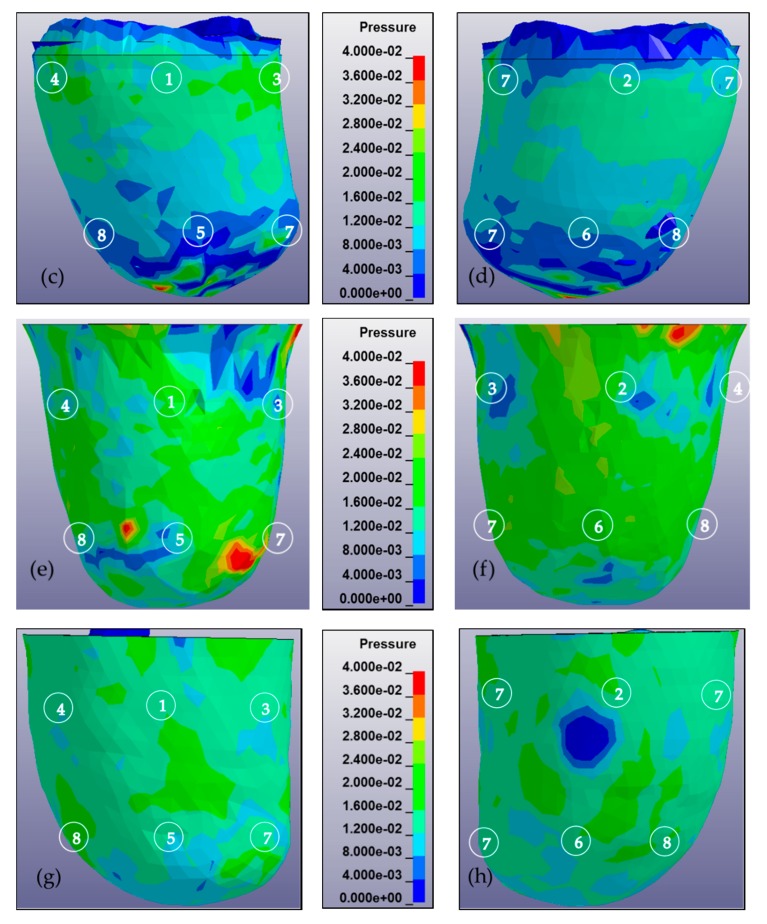
Pressure mapping of residuum during complete donning stages calculated from 0 to 40 kPa and observed from anterior and posterior views. The sequence of numbers on the mapping denotes the location of the sensor, i.e., AP, PP, MP, LP, AD, PD, MD, and LD. (**a**) Anterior view of residuum pressure mapping for subject A in MCCT socket. (**b**) Posterior view of residuum pressure mapping for subject A in MCCT socket. (**c**) Anterior view of residuum pressure mapping for subject A in UCLA socket. (**d**) Posterior view of residuum pressure mapping for subject A in UCLA socket. (**e**) Anterior view of residuum pressure mapping for subject B in MCCT socket. (**f**) Posterior view of residuum pressure mapping for subject B in MCCT socket. (**g**) Anterior view of residuum pressure mapping for subject B in UCLA socket. (**h**) Posterior view of residuum pressure mapping for subject B in UCLA socket.

In addition to the pressure measurement occurring in the eight locations of the sensors, a pressure mapping occurring inside the socket was also observed. Figure 6 shows the pressure mapping occurring in the anterior and posterior views. The number in the mapping indicates the location of the sensor during the experiment.

A high correlation coefficient was generally observed in most of the models compared. Most of the simulation results were lower than the experiment data. In the subject A-MCCT socket case, the total pressure was recorded as 166.72 kPa in the simulation (FEA), whereas in the experiment (EXP), the number was 18.45% higher. In the UCLA case, the total pressure was recorded as 84.11 kPa, and for the experiment, a 44.19% increment was achieved. The pressure occurring in the AP was the highest for both FEA and EXP for the MCCT socket case, whereas the pressure in the MP was higher than in the UCLA socket case for both the FEA and EXP results. A comparison of the pressure between the distal and proximal areas in the simulation was made for subject A, whereas the pressures in the distal and proximal areas in the MCCT case were recorded as 78.53 and 118.95 kPa, respectively; and in the UCLA case, the pressures for these areas were recorded as 36.68 and 45.43 kPa.

In the subject B-MCCT socket case, total pressures of 148.84 and 183.11 kPa were recorded for the FEA and EXP, respectively. In the UCLA socket case, the total pressure was recorded as 127.53 and 159.41 kPa for the FEA and EXP, respectively. In the MCCT socket for the subject B case, there was a significant difference in pressure detected in the LD position for the FEA and EXP. The highest pressure was detected in the LD, which occurred in the distal area for the MCCT socket case. However, in the UCLA socket case, higher pressure was detected in the AP, where it occurred in the proximal area. When a comparison was made between the pressure distribution in the proximal and distal areas in the MCCT-FEA, pressures of 69.16 and 79.68 kPa were detected. In the UCLA-FEA case, the pressures were recorded as 86.20 and 41.33 kPa for the distal and proximal areas, respectively.

## 4. Discussion

The tendency exhibited by the pressures obtained in the results for both the MCCT and UCLA sockets was almost the same for both subjects. This indicates that the shape profile of the two types of sockets ensured that the behavior of the residuum for each subject was almost identical.

**Figure 7:**
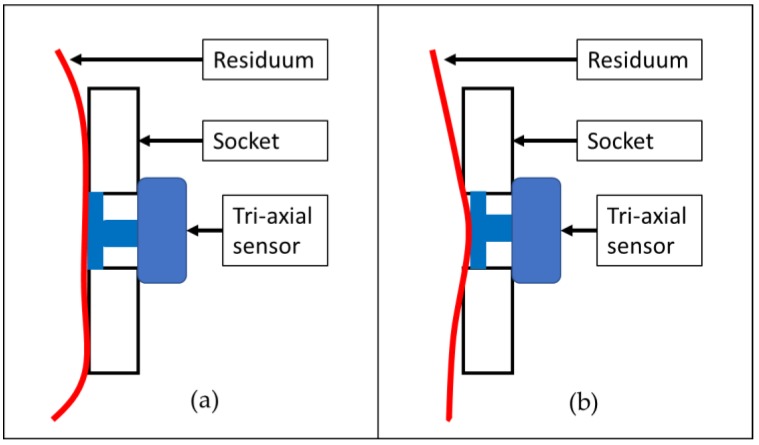
Illustration of contact surface between triaxial force sensor and residuum skin in a socket: (**a**) contraction of residuum with normal elasticity and (**b**) contraction of residuum with less elasticity.

Differences in the measurements of the FEA and EXP were observed in all locations at the sensors. However, the values exhibited a high correlation. In most cases, the measurements recorded in the FEA were lower than the EXP measurements. The reason for this phenomenon could have been the period during which the subject wore the socket. Both subjects had more than five years of history of wearing a socket, and the soft tissue of the residuum tends to reduce its elasticity. According to Sanders et al. [22], a mature residuum (>18 months postamputation) will continue experiencing daily fluctuations in volume and shape due to a reduction in muscle elasticity. A reduction in soft tissue elasticity contributes to maximizing the triaxial force sensor ability by increasing the contact force between the residuum skin and the force plate of the sensor, as illustrated in Figure 7. As for the FEA, the residuum model was set to be isotropic and homogenous, and the elasticity of the model remained constant.

The pressure observed in the proximal area was higher than in the distal area in most of the cases studied. The selection of different shapes between the residuum and socket model increased the tendency of the residuum geometrical deformation. During the complete donning stage, a high amount of volume deformation occurred in the proximal area because the prosthetic socket was created using a small cross-section in the proximal area to allow the socket to fit the subject residuum well [11]. The shrinking of the residuum increased the pressure occurring in the proximal area.

In other areas of the residuum that were not measured by the sensor, the distribution of the pressure was clearly observed in the FEA results. The results showed that the pattern of the pressure distribution on the surface was almost identical in both the MCCT and UCLA sockets. However, the amount of pressure occurring in the UCLA socket was higher compared to that of the MCCT socket in most cases.

Although a considerably realistic 3D model of the residuum was created in this study, which distinguishes between soft tissue fat and muscle parts, the separation of the muscle type of the residuum and its precise geometry is said to be more realistic and to enable an internal pressure analysis to investigate pressure ulcers and deep tissue injury (DTI) in an accurate environment [23]. The relative motion between the fat and muscle parts can be considered by enhancing the model in a realistic manner.

## 5. Conclusions

In this study, the pressure distribution profile of two subjects wearing UCLA and MCCT sockets was analyzed through a utilization of the finite element method (FEM). The analysis results showed that the measurement in the FEA exhibited a high correlation with the actual pressure distribution inside the socket during a standing phase. Although not all FEA measurements achieved results similar to the EXP, the results were considered promising because only a small average error (<10%) was determined during the FEA–EXP comparison. This error may have been caused by an abnormality in the actual residuum features, where scars and tissue injury in the actual residuum skin were not measured during the simulation. Furthermore, the mechanical properties used in the simulation were considered to be general, whereas the geometrical shape of the residuum was based on a subject-specific profile.

A comparison of the results of the proximal and distal areas showed that the pressure distribution on the surface of the proximal area in both the MCCT and UCLA sockets was higher than in the distal area. The results showed that the prosthetic socket was designed to minimize the pressure in the distal area such that the postoperation wound and scars in the residuum would not be affected, while at the same time the pressure in the proximal area was maximized to make the socket properly fit the subject’s residuum [11].

The main objective of this study, which involved an estimation of the pressure distribution profile using the FEM, was achieved. The results of the current study along with those of previous research studies indicate that the FEM is a suitable method for investigating residuum deformation behavior by measuring the pressure developed inside the socket. The pressure distribution profile helps the prostheses estimate the comfortability level of subjects while they are wearing the device. Thus, an adjustment can be made to the socket design before it has been fabricated. Although the calibration of a 3D model has been previously conducted [24], the details, particularly the scar from the post-treatment at the distal end, were not highlighted. In a future study, increasing the number of subjects will be our top priority to enhance the statistical analysis and consider subject-specific material properties through advanced finite element (FE) model creation.

## Figures and Tables

**Table 1:** Mechanical properties of viscoelastic material.

Part	Density (kg/m^3^)	*C*_1_ (kPa)	*C*_2_ (kPa)	*S*_1_	*S*_2_	*T*_1_ (ms)	*T*_2_ (ms)	*K* (MPa)
Skin	906	0.186	0.178	0.968	0.864	10.43	84.1	20
Fat	906	0.19	0.18	1	0.9	10	84	20
Muscle	1051	0.12	0.25	1.2	0.8	23	63	20
